# After cell death: the molecular machinery of efferocytosis

**DOI:** 10.1038/s12276-023-01070-5

**Published:** 2023-08-23

**Authors:** Byeongjin Moon, Susumin Yang, Hyunji Moon, Juyeon Lee, Daeho Park

**Affiliations:** 1 School of Life Sciences, Gwangju Institute of Science and Technology, Gwangju, 61005 Korea; 2 Cell Mechanobiology Laboratory, Gwangju Institute of Science and Technology, Gwangju, 61005 Korea

**Keywords:** Apoptosis, Time-lapse imaging

## Abstract

Cells constituting a multicellular organism die in a variety of ways throughout life, and most of them die via apoptosis under normal conditions. The occurrence of apoptosis is especially prevalent during development and in tissues with a high cellular turnover rate, such as the thymus and bone marrow. Interestingly, although the number of apoptotic cells produced daily is known to be innumerable in a healthy adult human body, apoptotic cells are rarely observed. This absence is due to the existence of a cellular process called efferocytosis that efficiently clears apoptotic cells. Studies over the past decades have focused on how phagocytes are able to remove apoptotic cells specifically, swiftly, and continuously, resulting in defined molecular and cellular events. In this review, we will discuss the current understanding of the clearance of apoptotic cells at the molecular level.

## Cellular housekeeping: clearing away dead cells

Vast numbers of cells die every day in a healthy adult and are cleared away in a process called efferocytosis in which ‘phagocyte’ cells engulf and degrade the remains. Daeho Park and colleagues at Gwangju Institute of Science and Technology in South Korea review the current understanding of efferocytosis at the molecular level. Research over several decades has discovered many of the molecules involved in the multiple stages of efferocytosis, which include finding, recognizing, engulfing, and degrading a wide variety of materials, but significant details remain to be uncovered. Defects in efferocytosis are implicated in many diseases, including autoimmune conditions, atherosclerosis, airway inflammation, and colitis. A better understanding of the multistep molecular processes of efferocytosis could open new avenues toward treatments for a variety of immunological and metabolic diseases.

## Introduction

Apoptosis is an essential process that occurs in multicellular organisms to eliminate unwanted cells, such as superfluous cells produced during development, aged cells that have lost their function, cells that can develop into cancer, damaged cells, and cells infected with viruses or bacteria, throughout life^1^. Through apoptosis, multicellular organisms can achieve developmental integrity, maintain tissue homeostasis, and protect themselves from the spread of infection^2^. However, inducing cell death of unwanted cells alone cannot accomplish these tasks. The dead cells must eventually be removed, as they can induce inflammation themselves. Removal of apoptotic cells, known as efferocytosis, is the final step of apoptosis^3^. It is known that hundreds of billions of cells undergo apoptosis in a healthy human body daily. However, apoptotic cells are rarely observed, even in tissues with a high cellular turnover rate^4^. For example, although most developing thymocytes in the thymus undergo apoptosis, apoptotic thymocytes are seldom seen in the thymus because they are swiftly and continuously removed^5,6^.

Efferocytosis is generally referred to as the phagocytosis of apoptotic cells because it involves the ingestion of extracellular substances^7^. Phagocytosis is the process of taking in extracellular particles larger than 0.5 µm in diameter by cells called phagocytes. In immunology, the term specifically refers to the ingestion and removal of bacteria and other pathogens within membrane-bound vesicles called phagosomes^8^. The common usage of the term phagocytosis for both pathogens and apoptotic cells may be confusing to individuals outside the field, as the immune response induced after phagocytosis of pathogens differs completely from the response after the engulfment of apoptotic cells. Pathogen phagocytosis often triggers proinflammatory responses, while engulfment of apoptotic cells generally induces anti-inflammatory responses or is immunologically silent^9,10^. Therefore, to avoid confusion, the term efferocytosis, which means "taking to the grave" or “burying,” was proposed instead of phagocytosis of apoptotic cells and is now used in the field^3^. Efferocytosis can be divided into four steps: recruitment of phagocytes to apoptotic cells, recognition of apoptotic cells by phagocytes, internalization of apoptotic cells into phagocytes, and degradation of apoptotic cells in phagocytes^11^. In this review, we will discuss the molecular mechanisms underlying each of these steps in efferocytosis. In addition, calcium signaling holds considerable importance in a wide range of cellular processes, and efferocytosis is no different. In the later part of this review, our focus will be on calcium signaling during efferocytosis and elucidating its molecular-level influence on each individual step of efferocytosis, which has not been previously reviewed.

## Apoptotic cell generation and the type of phagocytes

Our body is made up of ~37 trillion cells, and it is estimated that ~300 billion cells undergo apoptosis every day to eliminate superfluous, aged, and damaged cells, which ultimately maintain homeostasis. This is an astonishing number, representing 1% of the cells that make up our body^10,12,13^. Apoptosis occurs in various sites, especially in areas with a high cellular turnover rate, such as the thymus, bone marrow, mammary gland, and spleen. In the thymus, 95% of thymocytes undergo apoptosis during T-cell development, with only a small number of cells making it through key checkpoints^6^. In the bone marrow, a subset of excess immature B cells survives through a selection process during B cell development^14^. The mammary gland is also a site where apoptosis is actively occurring. After lactation, mammary epithelial cells undergo apoptosis^15,16^. Aged cells in need of replacement also undergo apoptosis. For example, neutrophils, whose lifespan is approximately 24 h, undergo apoptosis after one day. There are ~3000 to 7000 neutrophils in 1 µl of blood, and therefore, at least 18 billion neutrophils undergo apoptosis in an adult body every day^17–19^.

Phagocytes are a type of cell that defends the body by engulfing extracellular particles such as bacteria and dead or dying cells. There are two types of phagocytes, professional and nonprofessional, which are classified based on their level of effectiveness in phagocytosis^20^. The primary function of professional phagocytes is to remove apoptotic cells or exogenous particles such as bacteria. Macrophages and immature dendritic cells are examples of professional phagocytes that are specifically designed for phagocytosis and have the ability to quickly and continuously phagocytose targets^21^. In contrast, nonprofessional phagocytes, such as fibroblasts and epithelial cells, possess the ability to perform phagocytosis, but it is not their primary role^22–24^. Most cells in tissues or organs possess the ability to perform phagocytosis, although they are inferior to professional phagocytes in terms of rate and capacity. Fibroblasts and epithelial cells are examples of nonprofessional phagocytes^25^. Specialized phagocytes are a recently classified type of phagocytes whose primary role is not phagocytosis, but they have other functions similar to those of nonprofessional phagocytes, and they routinely remove apoptotic cells with phagocytosis, similar to professional phagocytes^26^. Retinal pigment epithelial (RPE) and Sertoli cells, as specialized phagocytes, remove the shed outer segments of photoreceptors in the retina and phagocytose apoptotic germ cells and the residual body of the sperm during spermatogenesis, respectively^27–31^. Additionally, astrocytes can be categorized as specialized phagocytes because they are phagocytic, mediate synapse elimination, and support neighboring neurons^32,33^.

## Molecular steps in efferocytosis

**Fig. 1:**
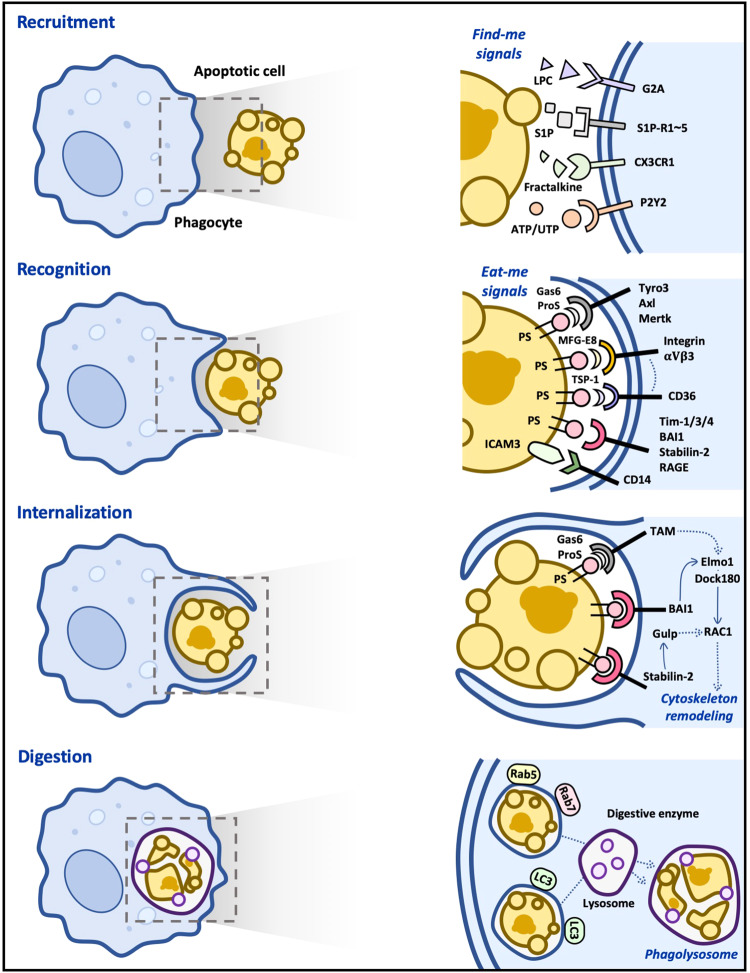
Molecular basis of efferocytosis. In the first step of efferocytosis, chemoattractants such as nucleotides and LPC released by apoptotic cells recruit macrophages. In the second step, the recruited macrophages directly or indirectly recognize apoptotic cells through ligand‒receptor interactions. This interaction leads to the activation of downstream signaling pathways, which ultimately results in the rearrangement of the cytoskeleton and internalization of apoptotic cells in the third step. In the final step of efferocytosis, internalized apoptotic cells form phagosomes and are degraded by lysosomal enzymes. LC3-associated phagocytosis (LAP) is more efficient in degrading apoptotic cells than non-LAP.

There are various types of cell death in our body, but most cells die through apoptosis during development and homeostasis^34^. These apoptotic cells are generally cleared in four steps (Fig. 1). The first step is to identify apoptotic cells. Apoptotic cells may neighbor phagocytes, but if not, phagocytes need to find apoptotic cells. At this stage, apoptotic cells are not passive but actively recruit phagocytes to them by releasing chemoattractants called “find-me signals”. In the second step, phagocytes recognize the apoptotic cells that are adjacent to them. In this process, apoptotic cells are recognized by phagocytes through interactions between ligands exposed on apoptotic cells, called “eat-me signals”, and their receptors expressed on phagocytes. Since phagocytes distinguish cells to be phagocytosed from cells not to be phagocytosed through this step, it is one of the pivotal steps in efferocytosis. In this process, the best-known ligand exposed on apoptotic cells is phosphatidylserine, which is directly or indirectly sensed by various receptors on phagocytes. In the next step, apoptotic cells sensed by phagocytes are internalized into phagocytes. The ligand‒receptor interaction activates signaling pathways downstream of engulfment receptors, which primarily induces cytoskeletal rearrangement to ingest massive targets. In the final step, phagosomes with internalized apoptotic cells fuse to lysosomes to form phagolysosomes. Apoptotic cells undergo degradation by various digestive enzymes derived from lysosomes. We will discuss the key molecules involved in these steps in detail (Table 1).

**Table 1:** Molecules involved in efferocytosis.

Find-me signals	Receptor	Reference
LPC	G2A	^36–38^
S1P	S1P-R1 ~ 5	^39–41^
Fractalkine (CX3CL1)	CX3CR1	^42,43^
Nucleotides (ATP/UTP)	P2Y2	^44,45^
ICAM3	CD14	^48^
**Eat-me signals**		
Phosphatidylserine (PS)	BAI1	^71^
	Tim-1, 3, 4	^68–70,81,90–95,109^
	CD300b	^48^
	CD300f	^61^
	RAGE	^73^
	Stabilin-1, 2	^72,89^
	Integrin αVβ3/5 (MFG-E8)	^20,77^
	Axl, Mertk, Tyro3 (Gas6, Protein S)	^74,75,79–81,94,95^
	CD36 (TSP-1)	^76–78^
Calreticulin (CRT)	LRP1 (CD91)	^65^
ICAM3	CD14	^66,67^
Complement components C1q	LRP1 (CD91)	^64^
	MEGF10	^33^
	SCARF1	^48^
**Don’t eat-me signals**		
CD47	SIRPα	^57,58^
CD24	Siglec-10	^57,62,82^
CD31	CD31	^57^
MHC-I	LILRB1	^84^
PD-L1	PD-1	^83^
**Molecules involved in internalization and degradation**		
Elmo1, Dock180, Rac1		^85–88^
Gulp		^89^
Scar, WAVE, Arp2/3		^11^
Rab5, Rab7, LAMP1		^96,97^
LXR		^100,101^
PPAR		^101^
Ucp2		^102^
Slc2a1, Sgk1, Slc16a1		^106^
**Molecules involved in calcium flux in efferocytosis**		
Undertaker, Junctophilin-1		^110,111^
Orai1-Stim1		^107,111^
Drp-1		^110,112^
Crbn-Orai1		^113^

### Recruitment of phagocytes

Numerous apoptotic cells are produced every day, but few apoptotic cells are seen in a steady state. This absence is because efferocytosis is highly efficient and promptly removes apoptotic cells. If apoptotic cells and phagocytes are adjacent, finding apoptotic cells does not affect the efficiency of efferocytosis. However, when they are distant, the effectiveness of efferocytosis depends on the phagocyte’s ability to locate apoptotic cells. Phagocytes do not randomly search for apoptotic cells but move in a specific direction that is induced by molecules released by apoptotic cells. These molecules, known as “find-me signals”, act as chemoattractants, causing the chemotaxis of phagocytes toward apoptotic cells (Fig. 1). Interestingly, find-me signals not only direct phagocytes to the location of apoptotic cells but also enhance their ability to clear apoptotic cells and induce anti-inflammatory responses^4,35^.

Several find-me signals have been identified thus far. Fractalkine (CX3CL1), sphingosine 1-phosphate (S1P), lysophosphatidylcholine (LPC), and nucleotides (ATP and UTP) have attracted the most attention. LPC is the first find-me signal discovered, and along with S1P, it is one of the lipid find-me signals^36^. It was reported that LPC was produced by the cleavage of phosphatidylcholine by phospholipase A2 activated by Caspase-3 during apoptosis and suggested that ABCA1 might be crucial for LPC release from apoptotic cells. LPC released from apoptotic cells is recognized by G2A, a G-protein coupled receptor, and is known to induce cell migration from phagocytes to apoptotic cells^36,37^. However, further investigation is necessary to determine whether LPC acts as a find-me signal in vivo, given that the concentration of LPC used in a previous study was much higher than that found in apoptotic supernatants and plasma^38^.

S1P is another lipid find-me signal^39^. Although S1P was previously known to be released from apoptotic cells, its role in inducing phagocyte chemotaxis in efferocytosis was not clear^40^. Gude et al. reported that during apoptosis, sphingosine kinase 1 (SphK1) upregulates and increases the release of S1P from apoptotic cells. They also demonstrated that purified S1P induces phagocyte migration^39^. S1P receptors may be involved in phagocyte migration. However, the expression of several S1P receptors (1–5) in phagocytes makes it difficult to determine which receptor is responsible for phagocyte migration in efferocytosis. Moreover, similar to LPC, the concentration of S1P required to induce phagocyte migration is much higher than that found in apoptotic supernatants^41^. Further investigations are needed to determine whether S1P acts as a find-me signal in vivo.

The protein fractalkine is found in the cell membrane and is released from apoptotic B cells and neurons. During apoptosis, cells form membrane blebs, which are released in the form of microparticles. Fractalkine-associated microparticles released from apoptotic cells are known to induce monocyte chemotaxis toward apoptotic B cells^42,43^. Studies have shown that CX3CR1 receptors on phagocytes sense fractalkine and induce phagocyte migration toward apoptotic cells. However, fractalkine is limited in tissue distribution, as it is only expressed in a few cell types.

Nucleotides such as ATP and UTP also serve as find-me signals during efferocytosis^44^. Nucleotides are known to be released from both apoptotic and necrotic cells, but much smaller amounts of nucleotides are released from apoptotic cells in a regulated manner. It was shown that nucleotides released from apoptotic cells induced phagocyte chemotaxis in vitro and in vivo, and co-treatment of nucleotides with apyrase, an ATP diphosphohydrolase, impaired phagocyte chemotaxis and efferocytosis in vivo^44^. Although various purinergic receptors are known to recognize nucleotides, it was reported that ATP and UTP were recognized by the P2Y2 receptor, leading to phagocyte chemotaxis. The regulated-manner release of nucleotides from apoptotic cells is controlled by Panx1. During apoptosis, Caspase-3 and −7 cleave the C-terminal tail of Panx1 and generate an open conformation of Panx1, causing the release of nucleotides^45^.

There are various other find-me signals, such as ribosomal protein S19 and EMAP II, that have not been mentioned^46,47^. Further investigation is needed to determine whether they are secreted only from specific types of apoptotic cells. In addition, it will be interesting to investigate whether various find-me signals exhibit synergistic effects on phagocyte chemotaxis.

### Recognition of apoptotic cells

Phagocytes have another task to distinguish cells to be phagocytosed from cells not to be phagocytosed, even though phagocytes are recruited into the proximity of apoptotic cells by find-me signals. This recognition is achieved by the interaction between ligands on apoptotic cells, called eat-me signals, and their receptors on phagocytes. Apoptotic cells expose various ligands on the cell surface that are not expressed in live cells. These ligands on apoptotic cells are sensed by receptors on phagocytes indirectly by bridging molecules or directly (Fig. 1)^48^. In this section, molecules involved in the second step of efferocytosis will be discussed.

Various eat-me signals present on apoptotic cells have been reported thus far^4^. These include phosphatidylserine (PS), oxLDL, calreticulin, ICAM3, C1q, and Annexin I. Among them, PS has attracted the most attention due to its universal exposure to apoptotic cells and the drastic inhibitory effect on efferocytosis upon masking it^49,50^. Some phospholipids, such as PS and phosphatidylethanolamine, exclusively exist in the inner leaflet of the plasma membrane in live cells. This asymmetric distribution of phospholipids between the lipid bilayer is maintained by phospholipid flippases that translocate specific phospholipids, such as PS, from the outer leaflet to the inner leaflet of the plasma membrane^51^. The asymmetric distribution is disrupted through the inactivation of flippases and activation of scramblases during apoptosis, exposing PS on the cell surface^52^. It was reported that ATP11, a flippase, was cleaved, leading to its inactivation, whereas Xkr8, a scramblase, was also cleaved by caspases, leading to its activation during apoptosis, which eventually exposed PS on the surface of apoptotic cells^53,54^.

It seems that PS exposure on the cell surface is sufficient to induce phagocytosis of the target in some cases, but it is insufficient for phagocytosis of the target in other cases^53,55^. It was shown that even viable cells could be engulfed when they expressed PS^53^. In contrast, in some biological processes, such as platelet activation, live cells express PS^56^, but these cells are not engulfed. Additionally, forced PS exposure on live cells through a mutant of TMEM16F, a calcium-dependent scramblase, failed to phagocytose live cells^55^. One possible explanation for the failure of engulfment of PS-exposed live cells is that PS-exposed live cells possess signals not to be engulfed, called don't eat-me signals^57^. In efferocytosis, phagocytes must remove only apoptotic cells without phagocytosing healthy and normal cells even though they expose PS on the cell surface. This specificity is achieved by the balance of the two signals, the eat-me and don’t eat-me signals. Cells that should not be engulfed may possess both signals, but cells that should be engulfed present only eat-me signals, resulting in efferocytosis. Don’t eat-me signals include CD31, CD47, CD24, PD-L1, and MHC I^57^. A molecular mechanism by which the signals prevent phagocytosis appears to be shared. Upon binding of don’t eat-me signals to their receptors, phosphatases such as SHP-1 and SHP-2 are activated and dephosphorylate myosin II^58–63^. Overall, the presence of eat-me signals on target cells are required for phagocytes to engulf them.

As mentioned above, a number of receptors expressed on phagocytes sense the ligands on apoptotic cells. These are collectively called engulfment receptors and include lectins, LRP1, CD14, scavenger receptors such as CD68 and SR-A, DD1α, and receptors recognizing PS^48^. Due to the importance of PS as a ligand on apoptotic cells in efferocytosis, this section will mainly discuss engulfment receptors that recognize PS. The engulfment receptors that recognize PS can be divided into two types based on how they recognize PS. One group recognizes PS directly, also called PS receptors, while the other group recognizes PS indirectly by bridging molecules^11^. This mode of recognition of apoptotic cells, indirectly by bridging molecules or direct recognition of apoptotic cells, also applies to other engulfment receptors that do not sense PS. For example, LRP1 indirectly recognizes apoptotic cells via calreticulin, whereas CD14 binds to ICAM3 exposed to apoptotic cells^64–67^. It has been reported that Tim-1, Tim-3, Tim-4, BAI1, Stabilin-2, and RAGE are engulfment receptors that directly recognize PS^68–73^, whereas members of the TAM family, integrins such as αVβ3 and αVβ5, and CD36 are engulfment receptors that indirectly recognize PS on apoptotic cells by bridging molecules^13,74–77^. CD36 is also known to directly bind to oxidized PS or oxidized LDL^78^. TSP-1, Gas6, protein S, and MFG-E8 function as linkers connecting PS with CD36, TAM members, and integrins, respectively^76,77,79,80^. In particular, Gas6 and protein S are well characterized. Gas6 binds to Mertk and Axl among the TAM family members and has a high binding affinity to Axl, while protein S binds to Mertk and Tyro3^81^. Engulfment receptors for eat-me signals are pro-phagocytic and promote efferocytosis upon binding of ligands to engulfment receptors by the mechanism described in the next section. In contrast, don’t eat-me signals engage anti-phagocytic receptors that prevent the engulfment of apoptotic cells, and several such receptors have also been identified^57^. SIRPα and Siglec-10 are representative receptors for the don’t eat-me signals CD47 and CD24 as ligands, respectively^58,82^. It was shown that SIRPα recruited SHP-1 upon CD47 binding, causing dephosphorylation of myosin II and, thus, disruption of contractile force to ingest apoptotic cells. Additionally, PD-1 and LILRB1 are also known as anti-phagocytic receptors for the don't eat-me signal^83,84^.

### Internalization of apoptotic cells

A pivotal molecule for the internalization of apoptotic cells in efferocytosis is Rac1, a member of the Rho family GTPase^85^. Its activation results in actin polymerization, which is required for the internalization of apoptotic cells (Fig. 1)^86^. Studies in *C*. *elegans* have identified two evolutionarily conserved signaling pathways for the phagocytosis of apoptotic cells: CED-2/CED-12/CED-5 and CED-1/CED-6, which activate CED-10, the Rac1 ortholog in *C*. *elegans*^87^. Engagement of eat-me signals with their receptors activates downstream signaling pathways that ultimately activate Rac1, inducing cytoskeletal rearrangement to engulf the target^88^. Although various engulfment receptors have been identified, not all signaling pathways downstream of engulfment receptors inducing cytoskeletal rearrangement are clear. However, the signaling pathway downstream of BAI1, one of the PS receptors, is relatively well established^71^. BAI1 was found to be a protein that interacts with Elmo1 (a mammalian ortholog of CED-12), acting as a bipartite GEF together with Dock180 (a mammalian ortholog of CED-5) for Rac1. BAI1 senses PS on apoptotic cells through the thrombospondin type 1 repeats of the extracellular domain, which leads to the activation of the Elmo1-Dock180-Rac1 signal module, causing actin cytoskeletal rearrangement^71^. Another PS receptor, Stabilin-2, is also known to interact with Gulp and activate Rac1 to promote efferocytosis^89^. In contrast, the signaling pathway downstream of Tim-4 remains elusive. Since Tim-4 without the cytoplasmic tail is still able to promote efferocytosis, it was considered a tethering receptor without direct signaling^90^. Tim-4 secured apoptotic cells on phagocytes, and then other engulfment receptors, such as integrin, generated signals to ingest the apoptotic cells, called two-step engulfment^91,92^. In addition, it was shown that tethering receptors could promote efferocytosis without biochemical interaction with their co-receptors, but Tim-4 biochemically interacted with its co-receptors, such as Mertk^93–95^.

### Degradation of apoptotic cells

The final step in efferocytosis is the degradation of apoptotic cells in phagocytes. Once internalized, apoptotic cells form phagosomes, which become increasingly acidic by recruiting Rab5 and Rab7 sequentially, ultimately fusing with lysosomes containing the digestive enzymes required for apoptotic cell degradation^96,97^. In this process, LC3-associated phagocytosis (LAP) matures phagosomes more efficiently than non-LAP, leading to the more rapid degradation of apoptotic cells (Fig. 1)^48,98^.

Since efferocytosis involves one cell engulfing another, the intracellular contents, such as carbohydrates, lipids, proteins, and nucleotides in phagocytes, are doubled in the final stage of efferocytosis, which can be a heavy burden for phagocytes. Thus, phagocytes release some of these contents and reprogram their metabolism to maintain appropriate levels of intracellular contents^99^. For example, apoptotic cell-derived sterols and recognition of PS by engulfment receptors activate LXR and PPAR, respectively, leading to an increase in Abca1 expression levels and cholesterol efflux^100,101^. In addition, phagocytes reprogram their energy metabolism. Upon apoptotic cell engulfment, the mitochondrial membrane potential (MMP) increases in phagocytes. Simultaneously, Ucp2, a protein that leaks MMP, is upregulated, which dissipates protons across the inner membrane of mitochondria and lowers MMP. Thus, Ucp2 maintains the appropriate MMP in phagocytes, enabling them to continuously engulf other apoptotic cells during efferocytosis^102^. Continuous efferocytosis is also enhanced by arginine derived from apoptotic cells, which is metabolized to putrescine that activates Rac1 by stabilizing Dbl mRNA. Thus, phagocytes that engulf the first apoptotic cell can more efficiently ingest the subsequent apoptotic cells due to increased Rac1 activation^103^.

Reprogramming of energy metabolism appears to affect the anti-inflammatory response as well. Apoptotic cell-derived methionine and fatty acids facilitate the production of anti-inflammatory cytokines such as IL-10, PGE2, and TGF-β1^104,105^. Furthermore, it was recently reported that efferocytosis increased glycolysis while decreasing oxidative phosphorylation, resulting in lactate release and upregulated IL-10 expression. Increases in the levels of Slc2al, Sgk1, and Slc16a1, which are involved in glucose transport and lactate release during efferocytosis, contribute to these results^106^.

## Calcium signaling in efferocytosis

**Fig. 2:**
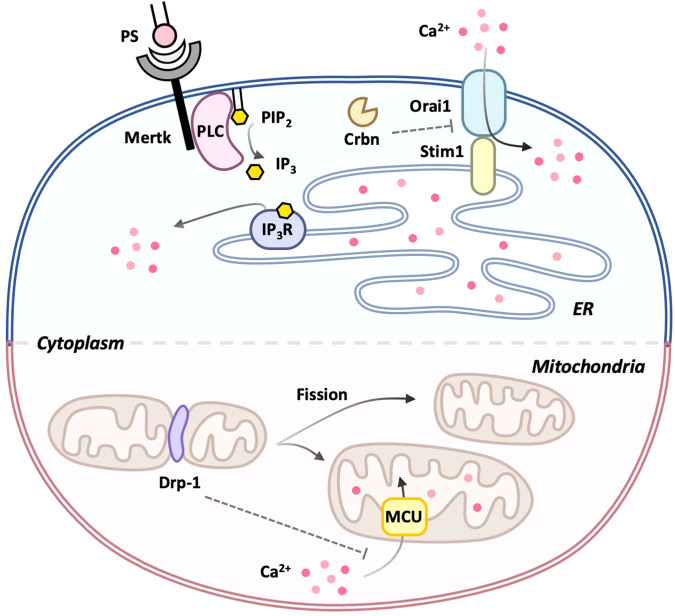
Calcium signaling in efferocytosis. Store-operated calcium entry (SOCE) is induced upon apoptotic cell stimulation. Mertk binding to PS on apoptotic cells activates the downstream signaling pathway, inducing the release of calcium from the ER and thus the interaction between Stim1 and Orai1, which ultimately causes calcium entry into phagocytes during efferocytosis. In addition, Drp-1 upregulation during efferocytosis induces mitochondrial fission, which impedes MCU-mediated mitochondrial calcium sequestration. This SOCE and impairment of mitochondrial calcium sequestration elevate the intracellular calcium level in phagocytes during efferocytosis.

Calcium is essential for various cellular processes, including efficient and continuous efferocytosis. Efferocytosis requires calcium both inside and outside phagocytes^107,108^. Depletion of either intracellular or extracellular calcium completely abrogates efferocytosis. Extracellular calcium is necessary for the recognition of apoptotic cells by phagocytes because a number of PS binding proteins, including PS receptors and bridging molecules, require calcium for their binding to PS^80,109,110^. Additionally, extracellular calcium acts as a provider, elevating intracellular calcium levels during efferocytosis^107^. Thus, the interruption of proteins involved in calcium influx impairs efferocytosis^111–114^. Studies have shown that Undertaker in *Drosophila* and Junctophilin in *C*. *elegans*, which link calcium channels at the plasma membrane to those of the endoplasmic reticulum, are required for efferocytosis. Disruption of Stim1 and Orai1, which are necessary for store-operated calcium entry (SOCE), also impairs efferocytosis^111,112^. Recent studies have reported that Mertk activates the PLCγ1-IP_3_R axis, causing the release of calcium from the ER and inducing the Orai1-Stim1 interaction and SOCE, leading to the elevation of calcium levels in phagocytes during efferocytosis^107^. Furthermore, Orai1, a calcium release-activated calcium channel mediating SOCE, is upregulated through the attenuation of Crbn-mediated ubiquitination, contributing to the elevation of intracellular calcium during efferocytosis (Fig. 2)^114^.

The elevation of calcium levels in phagocytes during efferocytosis is also the result of decreased calcium sequestration by mitochondria. A previous study reported that upregulation of Drp-1 during efferocytosis increases mitochondrial fission, which prevents MCU-mediated mitochondrial calcium uptake and thus increases the intracellular calcium level. Drp-1-mediated mitochondrial fission also facilitates the continuous removal of apoptotic cells by regulating vesicular trafficking and phagolysosomal degradation (Fig. 2). Therefore, one of the roles of intracellular calcium in efferocytosis appears to promote the degradation of apoptotic cells^113^. However, a better understanding of the role of intracellular calcium in efferocytosis requires further exploration.

## Concluding remarks

Apoptosis was discovered approximately half a century ago^115^, and swift and continuous efferocytosis is considered the final stage of this process. Research over the past several decades has discovered a variety of molecules involved in the multistep process of efferocytosis, including finding, recognizing, internalizing, and degrading apoptotic cells by phagocytes. Despite these long-standing efforts, several crucial questions about efferocytosis remain unanswered. In particular, the post-efferocytosis responses resulting from the different types of phagocytes and their efferocytosis receptors in tissues need to be further investigated. Additionally, since apoptotic cell degradation in phagocytes and the effects of their metabolites on phagocytes and neighboring cells have not been fully explored, further studies are needed.

Efferocytosis following apoptosis is an integral part of maintaining tissue homeostasis in multicellular organisms and is closely related to overall health. Indeed, defects in efferocytosis have been shown to be causative factors in numerous pathologies, including autoimmune diseases, atherosclerosis, airway inflammation, colitis, and others. Consequently, a better understanding of the multistep process of efferocytosis will provide new insights into the treatment of multiple immunological and metabolic diseases.
